# Neurocognitive responses to spatial design behaviors and tools among interior architecture students: a pilot study

**DOI:** 10.1038/s41598-024-55182-7

**Published:** 2024-02-23

**Authors:** Yaren Şekerci, Mehmet Uğur Kahraman, Özgü Özturan, Ertuğrul Çelik, Sevgi Şengül Ayan

**Affiliations:** 1 Interior Architecture and Environmental Design, Antalya Bilim University, Antalya, 07190 Turkey; 2 Akdeniz University, Interior Architecture, Antalya, 07070 Turkey; 3 Electrical and Computer Engineering, Antalya Bilim University, Antalya, 07190 Turkey; 4 Industrial Engineering, Antalya Bilim University, Antalya, 07190 Turkey

**Keywords:** Problem solving, Psychology and behaviour

## Abstract

The impact of emotions on human behavior is substantial, and the ability to recognize people's feelings has a wide range of practical applications including education. Here, the methods and tools of education are being calibrated according to the data gained over electroencephalogram (EEG) signals. The issue of which design tools would be ideal in the future of interior architecture education, is an uncertain field. It is important to measure the students’ emotional states while using manual and digital design tools to determine the different impacts. Brain-computer interfaces have made it possible to monitor emotional states in a way that is both convenient and economical. In the research of emotion recognition, EEG signals have been employed, and the resulting literature explains basic emotions as well as complicated scenarios that are created from the combination of numerous basic emotions. The objective of this study is to investigate the emotional states and degrees of attachment experienced by interior architecture students while engaging in their design processes. This includes examining the use of 2D or 3D tools, whether manual or digital, and identifying any changes in design tool usage and behaviors that may be influenced by different teaching techniques. Accordingly, the hierarchical clustering which is a technique used in data analysis to group objects into a hierarchical structure of clusters based on their similarities has been conducted.

## Introduction

Emotions have a substantial influence on human behavior and play a crucial role in social relationships, decision-making, and psychological health^1,2^. There are numerous applications of emotion recognition in education, teaching, marketing, and medicine^3–5^. In fact, emotions impact learning more than classroom design and teacher communication. Therefore, a comprehensive understanding of student behavior requires a firm grasp of emotions.

Interior architecture students must understand the emotional impact of design decisions made by designers who use different tools. Digital design tools have become prevalent, but many interior design professionals still use manual tools despite the practicality of digital tools for technical drawings. The design process involves understanding user needs, creating aesthetically pleasing and functional spaces, and developing proposals using 2D or 3D design methods. 2D design includes determining sub-space locations, relationships, sizes, circulation areas, and furniture placement. In space design, 2D design can be approached horizontally or vertically, while 3D design using models or digital tools allows for a holistic approach. Interior architecture education exposes students to the design process through courses covering design fundamentals, history, communication, presentation, construction, materials, and more. However, the curriculum's effectiveness in keeping up with evolving professional practices is uncertain, particularly regarding the use of manual versus digital tools. Therefore, understanding the emotional impact of different design behaviors and tools on interior architecture students is crucial.

Neurophysiological indicators such as heart rate, breathing, and brain activity are essential in determining emotional states as they are subjective experiences. Wearable EEG devices have made monitoring emotional states more accessible and more cost-effective than expensive medical equipment^6^. EEG studies have shown to be accurate and reliable in detecting emotional and mental states of individuals. The Emotive EPOC Neuroheadset and Muse headband are two popular wearable EEG devices that have been utilized in various studies^7^. Muse headband has been specifically used in experimental settings to determine mental states and emotional states of individuals^8,9^. EEG can also be used to evaluate user reactions such as long-term memory modulation, engagement, approach-avoidance determination, emotional arousal/valence, and sensory processing. The applications of wearable EEG devices are broad, ranging from improving brain-computer interfaces to enhancing care for neurological illnesses, advancing marketing research, and improving teaching methods.

EEG signals have been used to study emotion recognition, with different models and emotions being explored. The literature describes basic emotions and complex situations derived from combining several basic emotions, classified by various dimensional models. Russell's circumplex model is one of the most widely used models, where emotions are homogeneously distributed according to their valence and arousal values in a two-dimensional space^10^. Arousal refers to energy level, while valence indicates whether the energy is positive or negative. Different ways of calculating arousal/valence values exist in the literature, as well as various measures to understand emotional levels of users from EEG signals. The AW index, which is the frontal alpha asymmetry indicating motivation, has been used to determine if a person is approaching or withdrawing from a stimulus, and can also be applied in different design processes^11^.

EEG recordings generate extensive data that can improve emotion recognition by calculating arousal and valence values, facilitating the examination of associated emotions throughout time. Nevertheless, the process of conveying important knowledge from this vast amount of data to users is difficult without the participation of data specialists. Clustering analysis is crucial for extracting knowledge from huge data by recognizing pre-defined patterns within the EEG data and transforming these patterns into meaningful and actionable insights. By employing clustering methods like hierarchical clustering or k-means clustering, it is possible to uncover distinct patterns and relationships within the EEG data. This can assist in interpreting emotional states and enhance understanding for end-users ^12,13^.

The objective of this study is to provide a thorough examination of the emotional states and attachment experienced by interior architecture students during their design processes. This analysis will take into account the different approaches used, such as using either 2D or 3D tools, and encompassing both manual and digital methods. The study of emotional states will begin in the early stage, when the gathered emotional data will be used as unprocessed information. The generated findings from various design stages will be subjected to a detailed study utilizing the hierarchical clustering method. This analytical technique aims to make the data simply understandable for human interpretation while giving a brief summary of the different emotional states experienced during the design processes. In addition, the clustering analysis will thoroughly explore the emotions elicited by various methodologies, scrutinizing them from multiple angles such as time-related factors, population density, gender inequalities, and classification based on attendance at private or public colleges. The purpose of this inquiry is to determine the most efficient design techniques and technologies that can benefit students. The findings will provide valuable recommendations for interior architecture departments seeking to improve their teaching procedures. Furthermore, this study aims to investigate the possible discrepancies that may arise due to variations in teaching methods regarding the use of design tools and related behaviors among students studying interior architecture. This research intends to provide useful insights to instructional techniques in interior architecture education by clarifying these subtle distinctions.

### Related work

Design thinking is an analytical and creative process used by designers to invent solutions^14^. Initially, design was perceived as a "mechanical doctrine" based on systematic and rational methods^15,16^. However, design has since evolved as a self-consistent discipline with its own ways of knowing^15^. In the 1990s, there was increased questioning of the relationship between design and science^17^, resulting in the emergence of second-generation design methods that prioritize user or society-driven problem-solving approaches^15,16^. Design thinking tackles two types of problems: well-defined and ill-defined. Well-defined problems have a clear goal and a mechanical process for solutions, such as mathematical problems. Ill-defined problems, also known as design problems, are open-ended, complex, and incomplete, such as spatial design problems, and require questioning and solutions that may change over time^16–19^.

Design thinking varies across different fields, and the complexity of design problems leads to questions about how designers think while designing. Three paradigmatic approaches to measure the cognitive processes of design are design cognition, design physiology, and design neurocognition^15^. The first approach, design cognition, uses methods such as interview, questionnaire, scale, and observation. In contrast, the second approach, design physiology, focuses on physiological signals, such as eye movements and electro dermal activity. The third approach, design neurocognition, uses neuroscience techniques to analyze brain behavior during the design thinking process. Examples of studies that use a neurocognitive approach to understand the solution processes of under-defined problems in spatial design include Vartanian et al.'s studies on the effects of round or sharp-lined design approaches and ceiling height differences on aesthetic perception, Shin et al.'s study on the effects of direct and indirect lighting on brain cortex activity, Seitamaa-Hakkainen et al.'s study on the relationships between drawing, forming, skill learning, and the functional activities of the brain and its sub regions, Zhang et al.'s study on the effect of a designed landscape area and a natural landscape on human perception, and Vieira et al.'s study on neurophysiological activations of mechanical engineers and industrial designers based on design/problem solving^20–25^. Although the design cognition approach has become a valid experimental technique for exploring how the design process is cognitively understood, it is not sufficient to capture the conscious and unconscious design-oriented thinking process. However, design physiology and design neurocognition offer new ways to better understand human behaviors while designing, supplementing conventional design research. While it is challenging to develop appropriate, reliable experimental contexts in which to discover and quantify the precise interactions between design cognition and brain activity, EEG has a rich history of well-supervised trials that can be used in design studies.

2D orthographic drawings like plan, façade, and section drawings and 3D orthographic drawings like perspective expressions and physical models are used to communicate interior design information^26^. Transitioning from 2 to 3D or 3D to 2D helps build spatial design skills and mind-hand-eye coordination^27^. Spatial skills—2D and 3D technical expressions—are crucial for design drawing^26^. Since 1980, architects and interior designers have used computer-aided design (CAD). 3D modeling programs can be used as a design tool to test a product or space before it is built and as a presentation tool to make limitless copies and views of an object or space from a single 3D drawing. Computers draw faster and more precisely than ink and paper^28^. Researchers have wondered how using computer-aided design tools for space design affects creativity since some experts thinks that design needs right-brain creativity, however, CAD tools use the left brain. To draw a line or circle in AutoCAD, the most popular CAD tool used in architectural and interior design offices, the user types "Line" or "Circle" or "L" and "C". The left brain dominates thought processes because words are needed to input commands efficiently^29^. For the first primitive CAD programs and space designers used to traditional methods, converting a hand-drawn design into a technical expression in 2D and 3D was more suited for CAD, where rationality and left brain dominate. On the other hand, Brandon & McLain-Kark proved otherwise^30^. Their study found no significant difference in creativity between interior architecture students who did the course as a project course and those who used only manual tools or CAD programs. It is also unclear whether 2D or 3D computer-aided design tools are more creative than hand-drawn designs or whether students feel more comfortable with their designs or focus more on the design process.

Neurological study pioneers found that mice in "enriched" environments showed brain changes after two weeks compared to mice in "poor" environments. Social psychology shows that experiences alter people's thoughts^31^. The new generation, which was raised in a technologically advanced environment, has a distinct brain structure and way of thinking. Interior architecture programs that focus on space design try to include all design thinking methods and tools. After the pandemic and online course era, some interior architecture departments have eliminated hand drawing from their curriculum and focused on digital design behaviors and tools. Design thinking methods and tools for recent students have not been scientifically studied. This study examines the attachment, mood, and creativity of new interior architecture pupils using hand drawing or 2D and 3D CAD tools. This study seeks to identify today's students' best design thinking methods and tools. Design thinking methods and tools for interior architecture classes will be suggested as a result. It also examines whether gender or teaching methods affects design thinking and tools by using cognitive data analysis.

According to the findings of a number of different research^32,33^, the medial-frontal cortex is the area of the brain that is responsible for the processing of preferences, which is an essential component of emotion recognition. The medial prefrontal cortex, the nucleus accumbens, and the medial orbitofrontal cortex are the areas of the frontal brain that have been explicitly connected with preference. It has been demonstrated that activation in the nucleus accumbens is intimately associated with the decision-making behavior of people when they have to choose between different items and that preference inclinations expand as activity increases in the region of the medial orbitofrontal cortex. As a result of this, we came to the conclusion that while capturing EEG data for the study, we should concentrate on the medial-frontal cortex.

Suhaimi and colleagues analyze emotion stimuli, presentation method, research size, EEG hardware, machine learning classifiers, and classification approach in the state-of-the-art review. This state-of-the-art review recommends virtual reality stimuli for further research (VR)^7^. Tavares Da Silva group on the other hand presents a brainwave visualization system employing EEG and Eye Tracking to map people's emotional responses to audiovisual works, including attention and taste. The artifact was specified, implemented, and tested with 10 horror movie trailer viewers using Design Science Research. Participants received pre- and post-test questionnaires. The answers show emotional affiliation with the film, which may suggest a desire to see it in cinemas or a dislike of its theme/genre. In conclusion, this research suggests evaluating audiovisual works by considering unconscious emotional parts of subjective views of the seen content^34^. Dabas et al., presents a 3D emotional model to classify user feelings while watching musical videos. DEAP (Database for Emotion Analysis using Physiological Signals) is a standard dataset for investigating and interpreting human emotions using EEG signals are used in this research. EEG signals are captured as participants watch multiple videos and video liking emotions are classified according to arousal, valence and dominance. Valence-Arousal-Dominance space produced eight emotional states in this study as: comfortable, serene, bored, disgust, apprehensive, sad, astonished, and aroused^35^.

Menezes et al., also uses Russell's Circumplex Model to model affective states using electroencephalogram features just as we do in this study. They summed up how affect recognition can be used to improve VR environments for medical, educational, entertainment, and lifestyle applications. For the research, the DEAP dataset was used to get a set of EEG signals that had been labeled. Based on the Circumplex Model, Support Vector Machine (SVM) and Random Forest were used to group different affective states^36^. Basar et al. assessed emotional input with visuals and they use Common spatial patterns (CSP) technique. Experimental results and topographies on EEG data reveal that CSP spatial filtering implies the interaction between EEG bands, EEG channels, brain efficiency, and emotional input types^37^. Cao's group used the DEAP data set's EEG activity to classify subjects' emotions. Principal component analysis reduces the dimension of preprocessed EEG data to reveal the major emotional EEG patterns^38^. Al Nafjan's group employed deep neural network (DNN) for EEG-based emotion identification. They used PSD and frontal asymmetry parameters to train DNN to recognize human emotions from EEG signals^39^.

Upon reviewing the available research on emotion identification, it is clear that the DEAP dataset and several machine learning techniques are predominantly used. Nevertheless, our research utilizes three distinct design techniques to derive the arousal/valence and awareness signals from the data we gather in our laboratory with college students who are studying interior architecture. In addition, the application of Clustering analysis, a renowned machine learning technique, is used to analyze these signals. Approaches such as machine learning or statistical methods have been utilized to accomplish big data processing, such as EEG analysis. The research undertaken by Dabas et al. explores the utilization of EEG signals for the categorization of emotions. Their research focuses on exploring computational techniques for categorizing emotions based on EEG data. The study provides valuable insights into the utilization of electroencephalography (EEG) to categorize emotional states based on neural activity^35^. Zhang et al. propose a meticulous method for recognizing emotions based on EEG data by integrating clustering techniques with hybrid deep neural networks. The project will concentrate on enhancing the categorization of emotional states based on EEG signals by utilizing clustering methods and hybrid neural network designs. The text examines novel approaches for extracting features, with a focus on incorporating clustering techniques into deep learning frameworks to improve the accuracy of emotion recognition^40^. Furthermore, Ashgar et al. presents a novel method for selecting features in emotion recognition by employing deep feature clustering with multi-model neural networks. The study likely aims to improve feature selection methods by utilizing deep feature clustering, hence boosting the efficacy of neural networks in accurately identifying emotions from EEG data. It is probable that this adds innovative approaches to enhance the selection of distinguishing characteristics from EEG data in order to achieve more precise emotion detection^41^.

## Methodology

### Participants

This study included 32 participants (19 females, mean age 23.03), all of whom were senior interior architecture students. The students stated that they were in good mental health. As they have completed 3D modeling in most of the design studio classes, each student is quite familiar with at least one of the modeling programs. Furthermore, students are capable of using manual and 2D digital drawing tools.

To ensure the validity and reliability of the results of the study, participants were recruited from two different interior design departments, each with a different educational background. Specifically, 17 students from the interior design department of a state university, where education is more focused on manual drawing and physical model-making, and 15 students from the interior design department of a private university, where digital tools are more prominent in interior design education, were included in the study. By including participants from these two distinct backgrounds, the study aimed to compare the emotional responses of students to digital and manual design tools, and enhance the generalizability of the findings.

The inclusion of participants from two different backgrounds is particularly important as it helps to mitigate any potential biases and enhance the external validity of the study. It also enables researchers to investigate whether emotional responses to design tools and behaviors vary depending on the specific educational background of the student. Furthermore, it allows for a more comprehensive understanding of the emotional responses to design tools and behaviors, as it takes into account the unique experiences and preferences of students from different backgrounds.

Overall, the inclusion of participants from two different interior design departments with different educational backgrounds is a key aspect of the study, which adds to its robustness and generalizability. It enables researchers to investigate the emotional responses of students to design tools and behaviors across a wider range of backgrounds, and to explore how these responses may differ depending on the specific educational context.

The participants were asked to indicate whether they preferred starting their spatial design process with 3D design behavior, 2D design behavior with digital design tools, or 2D design behavior with manual design tools.

According to the survey results, 12.5% of the students (f = 4) preferred to start their spatial design process with 3D design behavior, which suggests that they may have preferred to visualize their designs in a 3D environment from the beginning. In contrast, 40.625% of the students (f = 13) preferred to begin with 2D design behavior and digital design tools, which may indicate that they are more comfortable using digital design software such as AutoCAD.

Interestingly, the majority of the participants (f = 15, 46.875%) reported starting their spatial design process with 2D design behavior and manual design tools. This suggests that they may prefer to sketch their ideas on paper before moving onto a digital 2D platform or a 3D model.

Overall, the survey results provide insight into the design preferences of the participants, indicating that a significant portion of them prefer to start their spatial design process with 2D design behavior regardless of the type of design tool they use. It also shows that there is a significant interest in using digital design tools among the participants, but it is not the only preferred method.

### Design of experiment and measures

**Figure 1:**
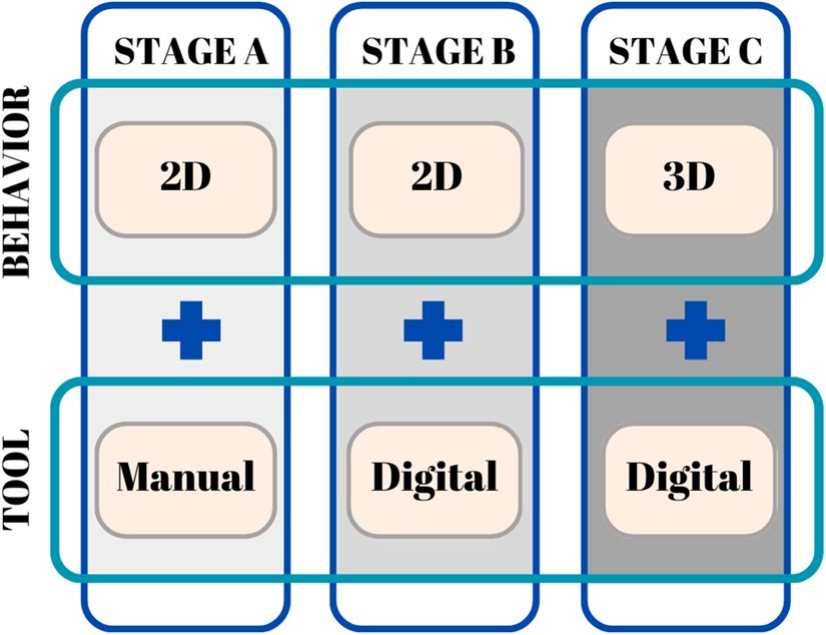
Experimental stages.

There are 3 stages to this experiment: Stage A, Stage B, and Stage C. One design tool and one design behavior are featured at each stage (Fig. 1). Students are tasked with planning each area of the house according to the state they are currently in using the provided design problems as guides. The residential space was picked because it is the most common type of space students meet in design classes and the type of space most people are born into and grow up with. In Table 1, we can see the design issues that have been established for each stage, as well as the design tool and behavior data that students are supposed to use.

**Table 1:** The requirements of the stages.

	Stage A	Stage B	Stage C
Space type	Residential-bathroom design	Residential-bedroom design	Residential-living room design
Scale	1/50	In 1/50 scale detail	1/1
m^2^	3.5 × 2 m = 7 m^2^	4 × 6 = 24 m^2^	6 × 5 m = 30 m^2^
Design tool	Millimeter paper, eraser, pencil, ruler etc	AutoCAD in the students’ own computer w/their familiar settings	One of the 3d modelling program in the students’ own computer w/their familiar settings
Design behavior	Manually design on 2D plan drawing	Digitally design on 2D plan drawing	Digitally design on 3D modelling
Design problem I	The room is entered through one of the 3.5 m walls	The room is entered through one of the 6 m walls	The room is entered through one of the 5 m walls
Design problem II	The entrance and the washbasin will face each other	There must be a window opening in one of the 4 m walls	There must be a window opening in one of the 5 m walls
Design problem III	–	The light will come from the left to the work area	Window openings cannot come behind the general seating elements
Design problem IV	–	–	The general seating elements should be able to see the view through the window, the TV and the fireplace
Needs in The Space	1 washbasin, 1 toilet, 1 shower area, 1 storage, 1 window/ventilation, 1 door, Floor covering	Double bed, 2 bedside tables, Headboard, Wardrobe, Work area (table, chair, bookcase), Floor covering	Seating elements for at least 5 people (armchairs, sofas, etc.), Window opening (you can determine the amount), Fireplace (view fire area), TV unit, Bookshelves and storage, Coffee-mid tables
Task	Not have to finish	Not have to finish	Not have to finish
Time	10 min	10 min	10 min

In shaping our experiment's design, we deliberately opted against incorporating three-dimensional physical model-making. This decision stems from the evolving trends in the professional and educational realms of interior design.

Contemporary interior design programs, including those attended by our participants, predominantly emphasize digital tools such as two-dimensional drawings and computer-aided design (CAD). Physical model-making, traditionally valued, is notably absent from the core curriculum of accredited programs.

Additionally, our EEG tool, Muse, is sensitive to movement. To maintain data accuracy, participants were asked to minimize movement during the procedure. The dynamic and physically demanding nature of three-dimensional model-making posed challenges, as it could introduce unwanted movement, potentially compromising the EEG data.

Considering time efficiency and participant engagement, we prioritized methods like 2D drawings and CAD modeling. These align with current industry practices, ensuring a seamless integration of our study within the contemporary design landscape.

In essence, our decision to exclude three-dimensional physical model-making was informed by the prevalent digital shift in design education, technological constraints, and a commitment to practical and efficient research methodologies.

The experiment was carried out in a room that was totally devoid of any ambient noise and was free of any outside influences. All participants may experience a carryover effect if these three steps are performed in the same order^42^. To avoid the carryover effect, these three phases will be administered to roughly the same number of participants in a different order. Table 2 depicts the order in which the participants performed these three steps during the experiment.

**Table 2:** Sequence of steps followed by participants in the experiment.

1. Step	2. Step	3. Step	# of Participants
A	B	C	5 students (1–5)
A	C	B	5 students (6–10)
B	A	C	5 students (11–15)
B	C	A	6 students (16–21)
C	A	B	5 students (22–26)
C	B	A	6 students (27–32)

### Ethical approval

All experimental protocols were approved by Akdeniz University Natural and Applied Sciences Scientific Research and Publication Ethics Committee dated 22.12.2022 and numbered 2022/18. All methods were carried out in accordance with relevant guidelines and regulations. Informed consent was obtained from all subjects and/or their legal guardian(s).

### Data recording, collection and preprocessing

Virtual Reality (VR) technologies have found substantial application in EEG studies related to design. Primarily, these technologies are employed when an immersive environmental stimulus is required^43–57^. This is particularly relevant when it is impractical to construct a 1/1 scale environment. In such instances, virtual environments emerge as optimal alternatives, surpassing traditional photographs in terms of realism.

In our study, we don’t need virtual or real environment as stimuli. Creativity and problem solving related studies doesn’t need a VR technology^58–62^.

Furthermore, eye-tracking and various methods for measuring physiological responses are predominantly utilized in studies involving VR and environmental stimuli^45,47,49,51,53,63^. In a specific VR-integrated design-based EEG study, despite the collection of eye-tracking data, it was not utilized in the study itself^56^. Modern tools like eye movement tracking systems measure programming technology effectiveness. They allow complex cognitive process analysis, unlike previous methods. An eye-tracking device and conventional knowledge level assessments were used by Katona to assess the readability of two semantically identical but syntactically divergent Language-Integrated-Query (LINQ) abstraction layer choices, comparing query and method syntaxes. Descriptive statistics show that eye movement tracking systems can help educators, developers, and researchers improve source code efficiency, maintainability, and advancement by studying complex cognitive processes like programming^64^.

**Figure 2:**
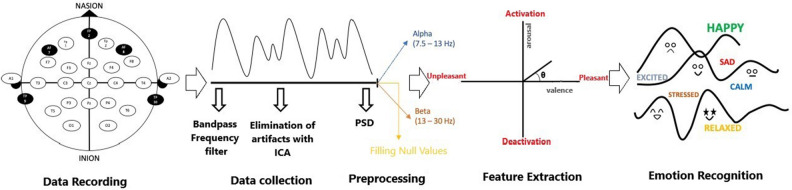
Steps of (**A**) data recording, (**B**) data collection and preprocessing, (**C**) feature extraction and (**D**) emotion recognition.

During the approximately ten minutes that each stage was being assessed by the thirty participants, EEG signals were captured every single second (Fig. 2A). Electrical activity in the brain was recorded using the Muse 2 headband, which records signals by making contact with the forehead of the subject^65^. The four channels utilized to record this activity were TP9, AF7, AF8 and TP10. Additionally, the data from the EEG was saved using the Mind Monitor app in the form of a comma-separated values, or CSV, file.

It is crucial to preprocess the EEG to remove noise and artifacts that impair the signal quality^66,67^. The noise was caused by several factors, including interference from power lines, electronic amplifiers, and other sources. Eye blinking and other joint motions are the primary sources of these changes, and their effects may be felt across several channels in the EEG data. Because of the potential problems caused by the noise introduced by these motions, Mind Monitor has incorporated automated techniques to remove artifacts. The EEG signals were segmented using a 0.5–50 Hz Bandpass filter to collect data for each of the five bands^67^ (Fig. 2B). Theta band (light sleep): 4–7.5 Hz; Alpha band (relaxation): 8–13 Hz; Beta band (active thinking): 14–29 Hz; Gamma band (deep thinking and learning): 30–45 Hz^9,10^. Delta band (deep sleep): 0.5–4 Hz; Theta band (light sleep): 4–7.5 Hz; Alpha band (relaxation): 8–13 Hz; Beta band (active thinking The Muse2 device does not completely follow the shape of everyone's forehead, hence there may be some cases of non-contact that result in null values in the dataset. These gaps in knowledge were filled using MATLAB's missing value filling technique^68^. We employed a moving average technique to estimate and replace the missing values, resulting in a more complete and reliable dataset for our research.

### Feature extraction

Each inquiry requires a unique approach to feature extraction based on its specific goals. The goal of this research is to provide a detailed synopsis of the range of feelings that are known to be experienced by students during the design process. Since arousal/valence values and an approach/withdrawal (AW) score are relevant for emotion recognition^69^, we will extract the frontal alpha and beta frequency bands to utilize in this analysis. In order to extract EEG frequency bands, the power spectral density (PSD) approach, commonly known as the Welch method, was used. The AW index and arousal/valence values are derived from the alpha and beta waves. Russell's two-dimensional model for classifying emotions uses the arctangent function to convert arousal and valence values to a single angular parameter (Fig. 2C).

In order to successfully tackle the issue of artifacts in EEG data and improve the precision of emotion classification, we have utilized Independent Component Analysis (ICA), a robust signal processing technique. Our research utilizes Independent Component Analysis (ICA) as a reliable method for removing artifacts from electroencephalogram (EEG) data, in accordance with established practices in the area. Prior research has extensively proven the efficacy of Independent Component Analysis (ICA) in artifact management and improving the quality of EEG signals. In their study, Plöchl et al. (2012) showcased the merging of EEG and eye tracking by employing Independent Component Analysis (ICA) to detect, describe, and rectify eye movement distortions in electroencephalographic data^70^. Burger and van den Heever also supported the utilization of Independent Component Analysis (ICA), in conjunction with a wavelet neural network, to eliminate electrooculographic (EOG) artifacts^71^. These studies confirm the dependability and effectiveness of ICA in dealing with distortions in EEG data.

In addition, our selection of ICA is consistent with the research conducted on the Muse headband, as published by López-Gil et al.^72^. This work aims to enhance the accuracy of emotion recognition using EEG data by combining synchronized biometric and eye tracking technologies. The study highlights the effectiveness of Independent Component Analysis (ICA) as a non-invasive and cost-effective method. Our modified PCA and ICA package incorporates ICA, which was influenced by Brian Moore's work in 2024^73^. Additionally, we have included fastICA, drawing from the findings of the "Classification of EEG Recordings by Using Fast Independent Component Analysis and Artificial Neural Network" study^74^. This contributes to the existing body of literature that acknowledges the effectiveness of ICA in processing EEG signals for emotion-related research in various contexts.

### Arousal and valence calculation

Characteristics of arousal and valence are used to recognize emotions (Fig. 2D). One way to think of arousal is as a quantitative measure of energy, while valence is a qualitative description of whether that energy is positive or negative ^5,75^. The electroencephalogram (EEG)'s frontal asymmetry can be used as a stand-in for arousal or valence. Also, valence and arousal are calculated with the help of a number of Eqs. ^69,75^ that are based on frontal asymmetry suggested in the literature^76–78^. Ramirez et al.^79^ came up with Eqs. (1) and (2), which we use in this study.

1 $$Arousal=Fronta{l}_{left\left(\alpha \right)}-Fronta{l}_{right\left(\alpha \right)}$$

2 $$Valence=\frac{Front(\beta )}{Front(\alpha )}$$

In order for Russell's model to work with the data, the normalizing approach had to be used. This procedure combines all of the numbers between -1 and 1 without losing any information^69^.

### AW index calculations

In cognitive neuroscience, the AW index is used to evaluate whether a person is leaning toward or away from a stimulus^80^. According to the frontal asymmetry theory^81^, positive and negative emotions (approach and withdrawal motivation) are processed in the frontal areas of the left and right hemispheres. According to Eq. (3), this index represents the degree to which the left frontal hemisphere is engaged in comparison to the right frontal hemisphere. Negative motivation (greater left frontal cortical activation) is associated with withdrawal behaviors^80,81^, while positive motivation (higher right frontal cortical activation) (approach behaviors) is associated with positive AW values.

3 $$AW=\frac{alfa\left(Front\_right\right) -alfa(Front\_left)}{alfa\left(Front\_right\right)+alfa(Front\_left)}$$

### Dimensional model of emotions

**Figure 3:**
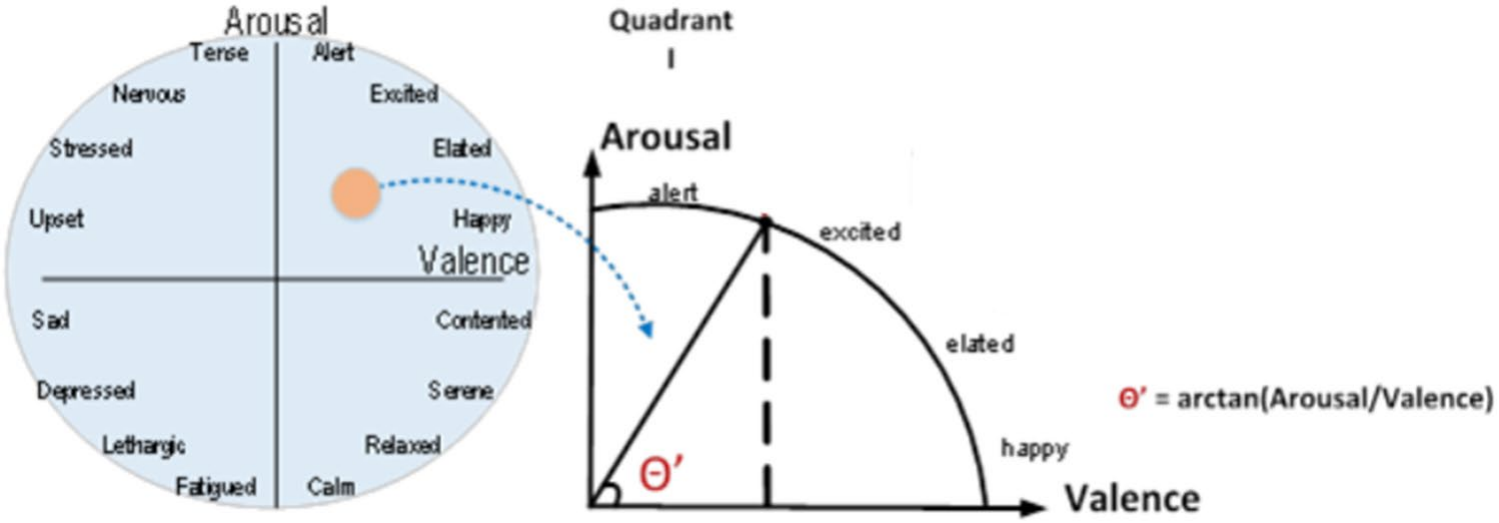
Emotion recognition from Russell’s circumplex.

Research into the accuracy of EEG-based emotion recognition was conducted using a variety of models and emotional states^82–84^. There have been numerous descriptions of both individual emotions^85^ and complicated circumstances^86^ that are the result of the combination of multiple emotions. Various dimensional models help to organize these feelings. One of the most widely used is Russell's circumplex model^84,87^. Emotions are homogeneously distributed according to their valence and arousal values in this two-dimensional model (Fig. 3).

Since emotional summaries were created with emotion labels occurred from EEG signals in the study, arousal/valence values need to be translated into emotion labels. The ratios of the arousal/valence values are converted to arctangent^82^ to determine the angle in Russell's circumplex. This angle transforms the EEG signal seen in the person to Russell's model. This angle is determined by using Eq. 4 to compute the angle between the horizontal plane and the ray that extends from the center to the points in the circle (which represents the intersection of the arousal and valence values^84^.

4 $$\theta =arctan\left(\frac{Arousal}{{\text{Valence}}}\right)$$

Figure 3 illustrates the representation of the angle that was calculated for each quadrant in the coordinate plane.

### Clustering analysis

Hierarchical cluster analysis, a data mining approach, involves partitioning data into clusters by grouping together related features. This strategy optimizes the similarity inside clusters and reduces the similarity between clusters. Our research involved the implementation of three distinct design processes, which were carried out by a group of 32 students. Students exhibit gender disparities and are enrolled in either private or public colleges. Arousal and valence values are calculated for each student. Using hierarchical clustering technique, our objective was to extract significant information from the extensive dataset of findings. The K-Means technique is the most prevalent algorithm utilized for clustering. This algorithm iteratively selects distinct center coordinates for the specified k clusters and assigns the nearest elements to their respective clusters. Each element is allocated to a cluster until the positions of the cluster centers remain unchanged. The number of clusters, k, is predetermined in the K-Means algorithm. Nevertheless, there are instances where it is impracticable to anticipate the quantity of like cells that may arise. For these situations, hierarchical clustering proves to be more advantageous. Put simply, hierarchical clustering requires active participation in the cluster creation process, allowing us to intervene at different levels. The key advantage of hierarchical clustering over the K-Means algorithm is the flexibility to influence clusters at various levels. In this study, we have employed hierarchical clustering. Clustering algorithms often rely on computing distances between observation data^84^. Hence, it is imperative to compute the distance between two places. The Euclidean distance is commonly employed in practical applications, and we have utilized it for our dataset. The Euclidean distance is determined for values of i and j ranging from 1 to n, and for k ranging from 1 to p, where p represents the number of variable as

$$distance\left(i,j\right)=\sqrt{\sum_{k=1}^{p}({x}_{ik}-{x}_{jk}{)}^{2}}$$

In hierarchical clustering, the outcomes of calculating the distance between each element of the data set are recorded in a matrix. When there is a need to partition n data into clusters, a distance matrix of size nxn is generated as a consequence of this process. Once the distance matrix is established, two comparable elements are merged to create a unified cluster. By combining each piece with one another, new sets are created. The distances between these clusters are computed, resulting in the creation of a distance matrix. This iterative process continues until all the pieces have been consolidated into a single cluster. Using a hierarchical tree structure, the elements are grouped together within a cluster. The dendrogram is the term used to refer to this tree-like structure. A dendrogram is a visual depiction frequently employed in hierarchical clustering analysis to demonstrate the organization of clusters formed during the clustering procedure. A dendrogram is a graphical representation that illustrates the hierarchical relationships between data points or groups in a tree-like structure. The outcomes of our clustering study and the corresponding dendrograms are displayed in the Results section.

## Results

**Figure 4:**
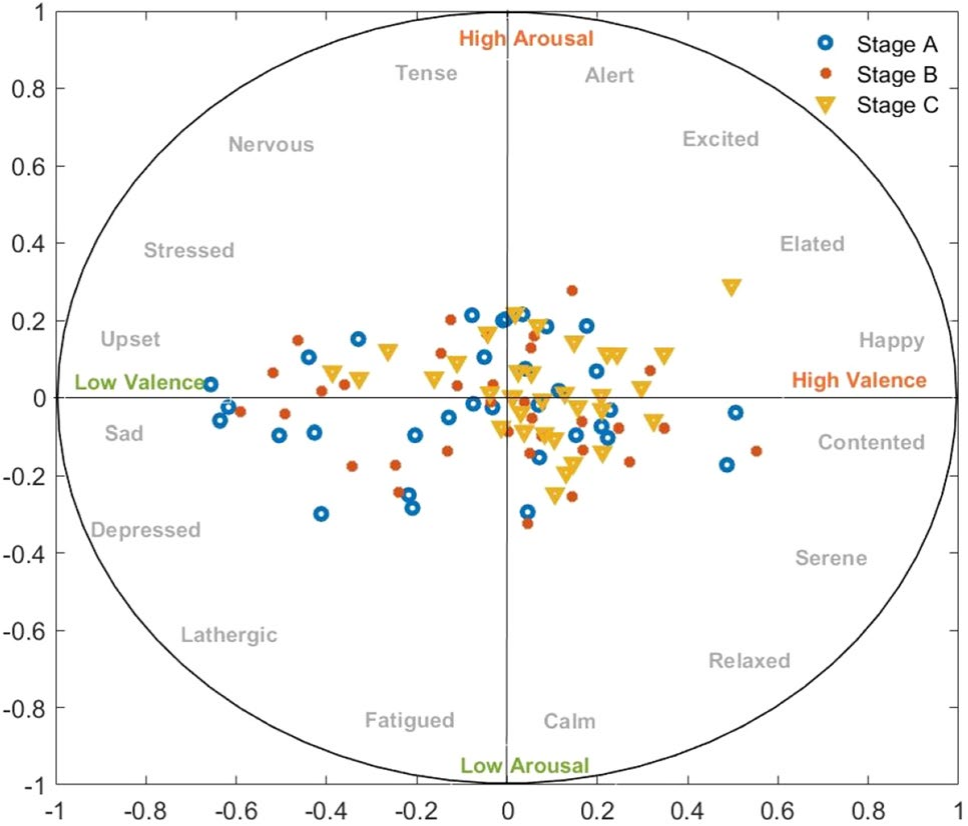
Representation of Two-Dimensional Valence and Arousal Chart depicting stages A, B, and C. Stage A is illustrated by a blue circle, Stage B by a red dot, and Stage C by a yellow triangle.

First, a two-dimensional valence and arousal chart was produced for the study. There are four quadrants in this graphic. The upper left quadrant is reserved for low valence, high arousal situations. This quadrant's emotions are generally associated with stress, tension, or anxiety. A significant concentration of Stage B data points in this quadrant indicates that individuals in this stage experienced unpleasant emotions mixed with high arousal, such as agitation or anxiousness. Fewer Stage A or C points in this location may imply less stress or agitation throughout these periods (Fig. 4).

The top right quadrant is reserved for high valence and arousal. This area displays high-energy positive emotions such as happiness or excitement. A dense cluster of Stage B in this quadrant could indicate that participants felt mostly happy and energetic feelings throughout this stage. Less representation of phases A and C may indicate a decrease in these very happy and aroused emotions or a transition to a calmer mood during these phases (Fig. 4).

The bottom left quadrant is reserved for low valence and arousal. This quadrant is commonly connected with melancholy, despair, or lethargy. The lack of Stage C observations in this quadrant shows that participants rarely experienced these low-energy and negative feelings during this phase. If Stages A and B are found here, it may imply that some individuals experienced low arousal and negative valence throughout these stages (Fig. 4).

The bottom right quadrant is reserved for people with strong valence but low arousal. With low arousal, this quadrant represents feelings of calm, tranquility, or satisfaction. The existence of period C here could indicate that individuals felt generally good yet tranquil feelings at this period. The lower intensity of Stages A and B in this quadrant suggests that other emotional states predominated during these stages (Fig. 4).

**Figure 5:**
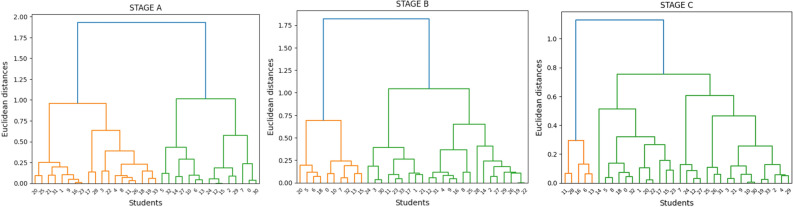
Dendrograms for each three stages.

Figure 5 depicts hierarchical clustering analysis for three separate rounds of a study, with the goal of finding discrete participant segments based on certain traits or replies. These graphic representations aid in determining the structure and relationships within the dataset, demonstrating how participants are categorized based on the similarity of their profiles.

The dendrogram for Stage A indicates the formation of a significant cluster at a greater Euclidean distance, suggesting a general commonality among a wide range of participants at this stage. The presence of smaller clusters at decreasing Euclidean distances shows the presence of more closely associated participant subgroups. This hierarchical structure suggests the dominance of a broad participant profile, with distinguishable sub-segments that share more strongly associated traits (Fig. 5).

In Stage B, the dendrogram reveals a more complex clustering arrangement. While there is a substantial cluster at a higher level of the hierarchy, several intermediate clusters are observed before this large cluster forms. This pattern could indicate a diversification of participant profiles, with subgroups defined by finer distinctions. The intermediate clusters preceding the final large aggregation may represent a stage in the study when participant characteristics are more variable, possibly due to the influence of various factors that became more pronounced during this phase (Fig. 5).

The dendrogram for Stage C shows a distinct clustering pattern, with clusters forming at lower Euclidean distances. This indicates more precise segmentation and improved homogeneity within each cluster. Compared to the previous stages, the main cluster forms at a significantly lower height, suggesting a consolidation of participant groupings into more uniform profiles (Fig. 5).

**Figure 6:**
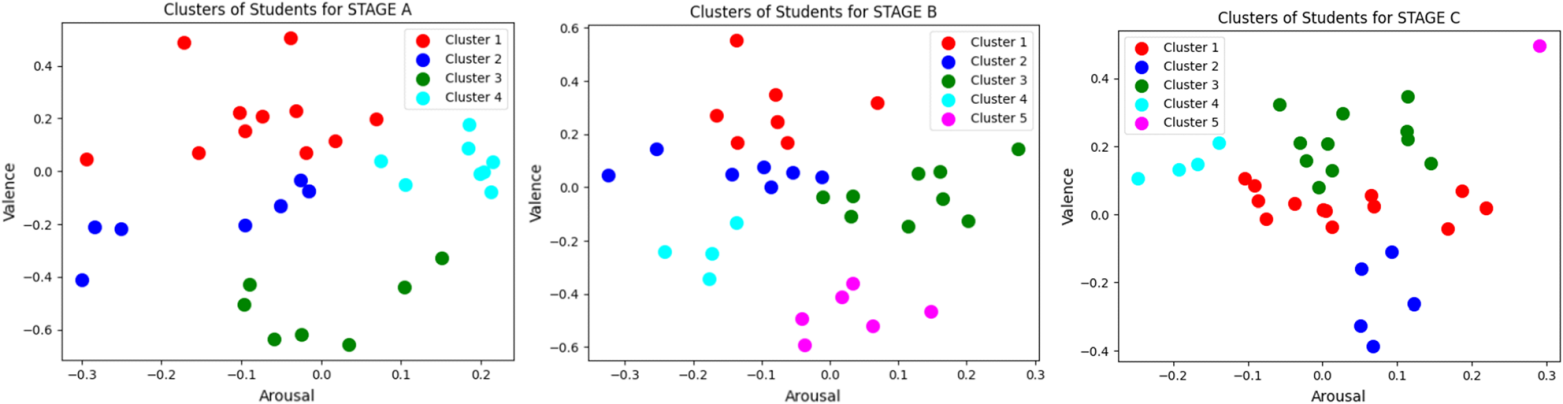
Scatter plot analyzes for each three stages.

Figure 6 depicts scatter plots for three stages of a study, designated as participant clusters. These plots show how participants are distributed across two metrics: valence and arousal. A different color represents each cluster.

For Stage A, four unique clusters are observed. Clusters 1 and 4 exhibit higher Valence values, suggesting happier emotions among these subjects. Cluster 2, representing neutral Valence and Arousal, indicates a balanced emotional state. Cluster 3 is situated in the lower Valence area, implying more negative emotions. The Arousal levels are relatively uniform, with no extreme outliers (Fig. 6).

Stage B features five clusters. The introduction of Cluster 5 and the changes in existing clusters suggest a dynamic emotional landscape among participants. Cluster 2 remains centrally positioned, indicating a continuation of neutral emotions and arousal. The spread of Clusters 3, 4, and 5 across Valence and Arousal suggests varied emotional responses (Fig. 6).

In Stage C, the scatter plot shows four clusters. The reduction to four clusters could indicate a convergence in emotional states. Clusters 1 and 3, with higher Valence, reflect more positive emotions, while Cluster 2 indicates lower Valence. The absence of Cluster 5, present in Stage B, may suggest a merging of emotional characteristics or a less distinct separation of emotional states (Fig. 6).

Pie charts for Stages A, B, and C show the percentage distribution of all participants' emotional states over four categories: Low Valence Low Arousal (LVLA), Low Valence High Arousal (LVHA), High Valence Low Arousal (HVLA), and High Valence High Arousal (HVHA).

**Figure 7:**
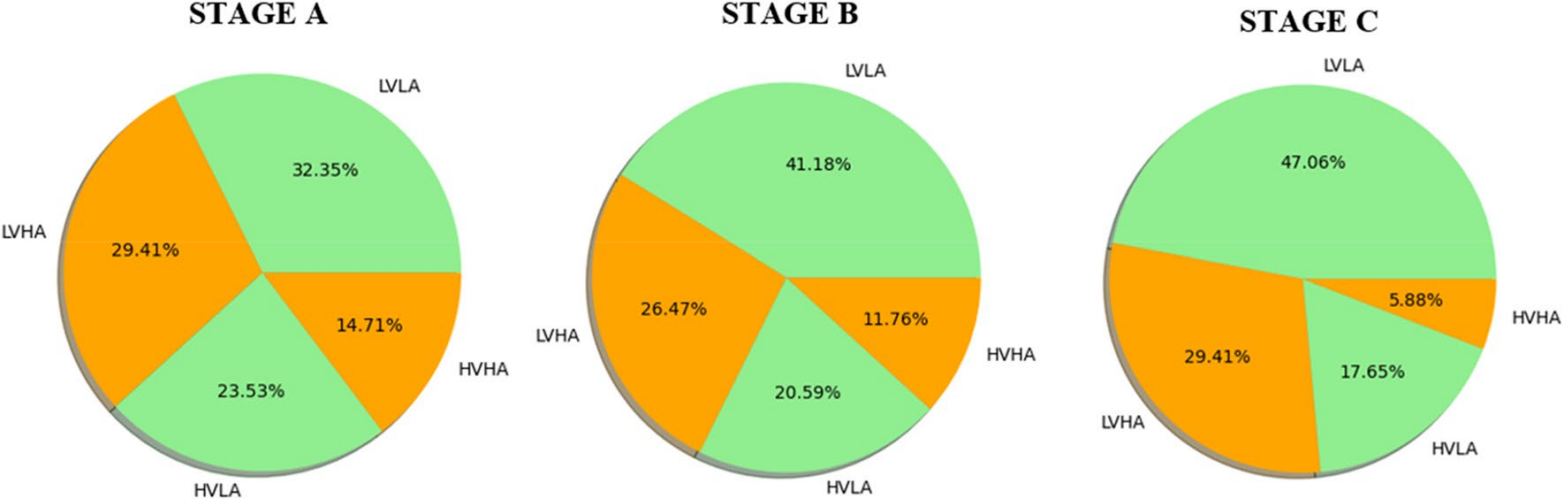
LVLA, LVHA, HVLA, and HVHA pies for each three stages. The visual representation indicates regions: LVLA (low valence low arousal), LVHA (low valence high arousal), HVLA (high valence low arousal), and HVHA (high valence high arousal).

The majority of the distribution in Stage A is in the HVLA quadrant, indicating that individuals had good and tranquil feelings. The next highest level is LVHA, which indicates that some subjects experienced negative feelings while being highly aroused. HVHA is the lowest, implying that there are fewer instances of positive high-energy emotions (Fig. 7).

Stage B shows a more dominant distribution in the HVLA quadrant compared to its random occurrence in the series of stages, indicating an increase in happy and tranquil feelings for participants in this stage. The presence of LVHA remained substantial, indicating that some participants in Stage B experienced negative but highly aroused emotions (Fig. 7).

There is a significant increase in the HVLA quadrant for participants in Stage C, reflecting a larger presence of happy and tranquil emotions compared to their random experience of this stage. The LVHA quadrant remains large, but HVHA has the smallest representation, indicating a further drop in positive feelings associated with high arousal for participants who experienced Stage C (Fig. 7).

**Figure 8:**
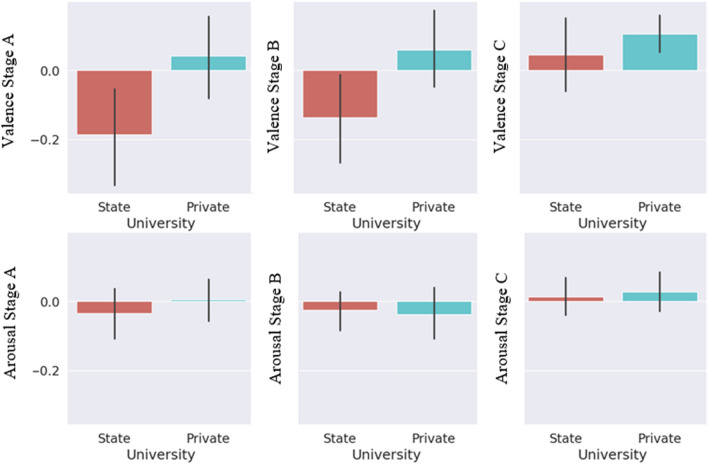
Comparison of the state university and private university students’ arousal and valence values for each three stages.

When we compare state and private university students, private university students had higher valence scores in Stage A than state university students, indicating more positive emotions during this stage. Arousal levels were higher in state university students than in private university students, indicating greater alertness or energy (Fig. 8).

Private university students had higher valence scores than state university students in Stage B, indicating more positive emotions. Private university students reported higher arousal levels than state university students at this stage (Fig. 8). In Stage C, private university students continue to report higher levels of arousal, indicating sustained higher levels of alertness or energy (Fig. 8).

Private university students consistently reported higher valence scores than state university students across all stages, indicating a general trend of experiencing more positive emotions regardless of stage. State university students had higher levels of arousal in Stage A, but private university students reported higher levels of arousal in Stages B and C.

**Figure 9:**
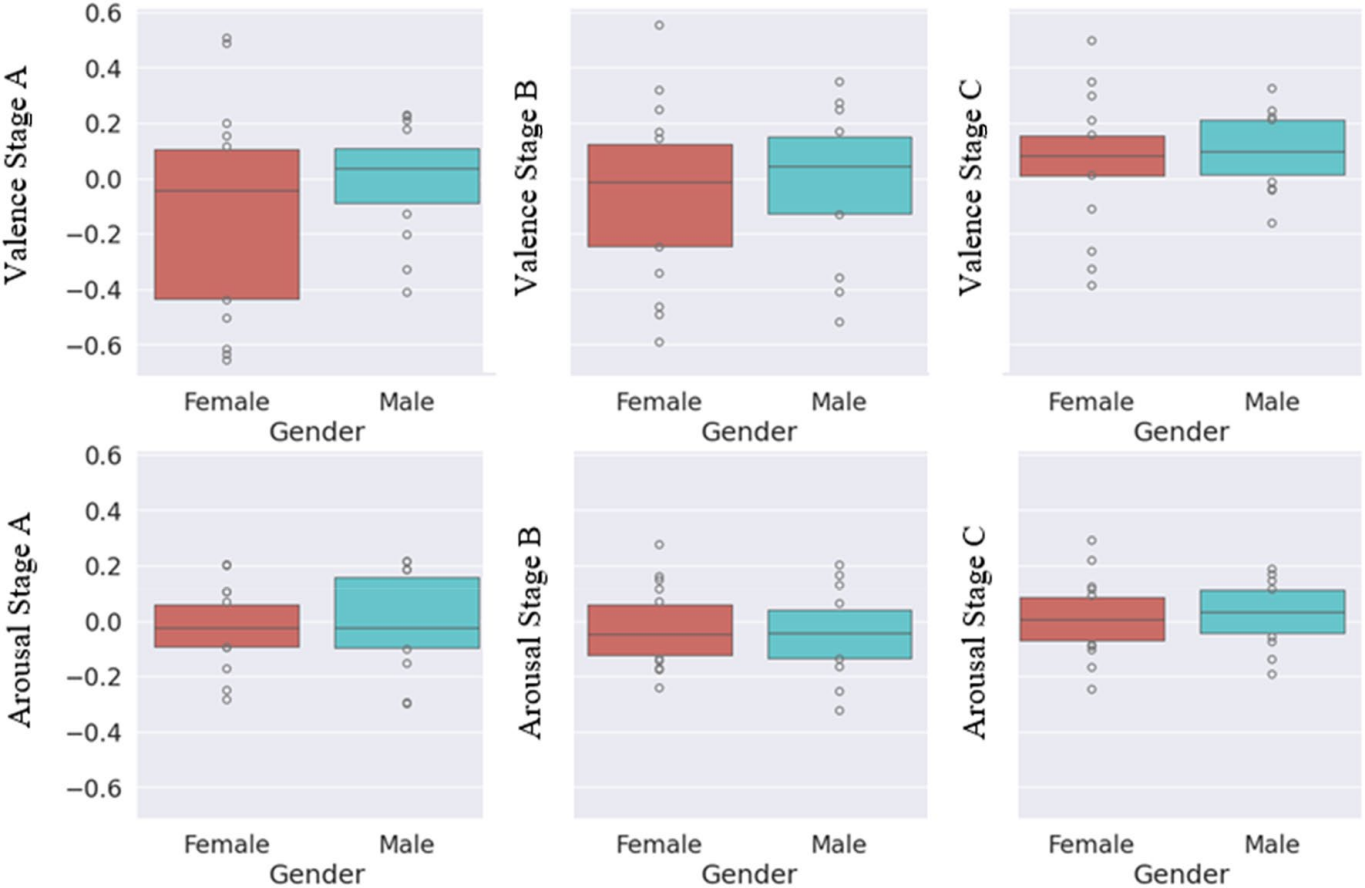
Comparison of the female and male students’ arousal and valence values for each three stages.

When female and male participants were compared in Stage A, male participants reported higher valence scores than female participants, indicating more positive emotions during this stage. Males had higher arousal scores than females, indicating a higher level of alertness or energy (Fig. 9).

The trend of males is reporting higher valence in Stage B. In this stage, males and females have similar arousal levels, but males report slightly higher levels (Fig. 9).

Male participants maintain higher valence scores in Stage C than female participants, indicating a consistent pattern of more positive emotions. Males have higher arousal levels, indicating that they were more energized or alert in this stage (Fig. 9).

Male participants consistently reported higher valence scores across all stages, implying that they experienced more positive feelings throughout the experiments. Males also demonstrated higher levels of arousal than females in each stage, showing a pattern of greater alertness or energy among male participants.

**Figure 10:**
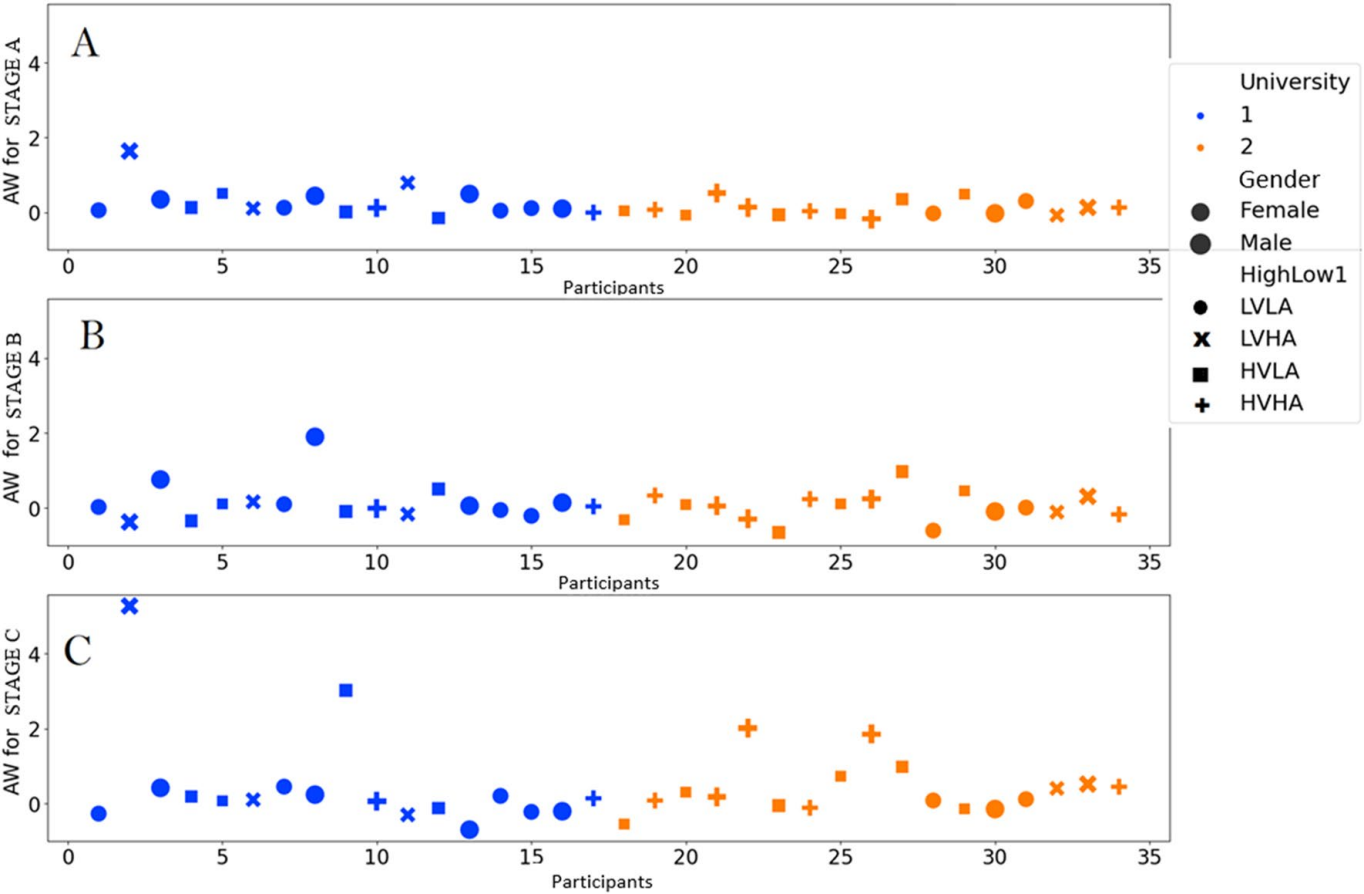
Participant distribution and clustering analysis across three stages, differentiating state (university 1) and private (university 2) institutions, emphasizing gender representation. Male students are depicted with larger symbols compared to female students. The visual representation indicates regions: LVLA (low valence low arousal—circle), LVHA (low valence high arousal—cross), HVLA (high valence low arousal—square), and HVHA (high valence high arousal—plus sign).

Figure 10 presents three scatter plots of AW values illustrating data from various stages of an experiment, designated as Stages A, B, and C. Negative motivation is associated with withdrawal behaviors and negative AW values, while positive motivation is associated with positive AW values. Each plot displays points that represent participants, categorized by university type (State or Private), gender (female or male), and the combination of Valence and Arousal levels. These combinations are High Valence High Arousal (HVHA), High Valence Low Arousal (HVLA), Low Valence High Arousal (LVHA), and Low Valence Low Arousal (LVLA).

During Stage A, the distribution of universities, both State and Private, is widely spread over the "AW" axis, lacking a discernible pattern to differentiate between the two types. Male students from private institutions are primarily located in HVHA, whereas female students from the same universities are predominantly positioned in HVLA. Participants from state institutions are evenly distributed across all four combinations of Valence and Arousal, with a little concentration in the LVLA category (Fig. 10A). The level of approach and withdrawal is nearly negligible at this time.

Stage B exhibits a higher level of concentration among participants along the "AW" axis, with a small clustering of individuals from private universities in HVLA and HVHA. Participants from state universities, particularly males, exhibit a higher occurrence in LVLA, indicating a comparable distribution pattern to Stage A. Stage B exhibits a greater number of positive AW values compared to Stage A, indicating that students are experiencing a higher level of attraction towards Stage B (Fig. 10B).

In Stage C, there is a notable disparity in the distribution, characterized by a prominent concentration of participants from state universities in LVLA, whereas their presence in HVHA is minimal. The majority of male students in private universities are centered in HVHA, whereas female students are mostly in HVLA. Stage C exhibits a noteworthy positive trend in AW values, particularly for private universities. This can be attributed to the fact that students predominantly utilize Stage C and are more acquainted with Stage C (Fig. 10C).

## Discussion

From childhood to adolescence and into higher education, drawing and writing with pencil and paper, and their combinations, become a reflexive action that does not involve any anxiety, stress, or other factors during their execution. Therefore, users do not experience any stress when engaging in manual drawing. However, drawing is an activity that requires the development of muscles, as it is performed through the muscles of the hand. Stage A showed a high concentration in the HVHA quadrant, indicating that participants experienced positive emotions combined with high arousal. Drawing by hand is a movement created solely by the person holding the pen, without any intermediary between the drawer and the drawing. As a result, simultaneous design is possible, and a sense of emotional unity can be achieved.

The trend towards computer-aided programs observed in Stage B is due to students' immersion in this environment. Computer-aided drawing programs are preferred because they are faster and easier to learn. Additionally, they allow for quick measurements, accurate proportions, copy and duplicate options, which can make them more attractive than manual drawing. In Stage B, the HVLA quadrant was more dominant, indicating that participants experienced calm and positive emotions. The "luxury of making mistakes" offered by the computer-aided drawing environment may have made the new generation feel positive and calm as they were born into the world of technology and interacting with computers are an ordinary part of their daily lives.

Stage C saw a significant increase in the LVHA quadrant, indicating that participants experienced negative emotions combined with high arousal. This is because 3D design behavior is inherently more difficult than 2D design behavior since it involves one more dimension. Besides, 3D design tools have more complex usage. It is understood that students struggle more compared to the other stages because they focus more on how to conduct the design tool rather than the design itself.

In all stages, male participants had more positive emotions because they showed higher valence values than female participants. This is a very interesting finding. Even though almost 95% of the interior architects were male in 1972, In recent years, approximately 70% of interior architecture students in interior architecture departments are women^89^. This shows that the interior architecture profession is mostly preferred by women. Despite this, it is interesting that male students showed more positive emotions during different design tools and behaviors in this study.

In all computer-aided stages which are Stage B and C, private university students have more positive feelings than state university students. On the contrary in manual stage which is Stage A, state university students have more positive feelings than private university students. Differences in the educational approach of the interior architecture departments of the two universities may actually play an important role here. While the state university interior architecture department focuses more on manual drawing, private universities switch to computer use in the early stages of education.

Furthermore, the results of our investigation open up possibilities for utilizing neurofeedback techniques in educational paradigms." Our study uses EEG monitoring to evaluate the emotional states of architecture students during different design processes. This allows us to understand the complex connection between cognitive activity and emotional experiences. Understanding this can be crucial in creating customized neurofeedback interventions with the goal of enhancing learning environments for design education. By utilizing EEG analysis to identify emotional reactions, educators and designers can implement real-time feedback mechanisms. This enables them to adapt instructional tactics or improve design tools to better align with students' emotional engagement. Exploring the integration of neurofeedback approaches in educational settings could enhance students' cognitive processes, creativity, and overall learning experiences in engineering and related subjects.

Prior research^90–93^ has demonstrated the promising potential of utilizing electroencephalogram (EEG) data to recognize and comprehend human emotions in diverse sectors. Shin, Maeng, and Kim emphasized the importance of utilizing several bio-signals to recognize interior emotions, emphasizing the usefulness of various physiological cues in deciphering emotional states^90^. In a similar vein, Saitis and Kalimeri showcased the effectiveness of using EEG and peripheral biosignals for multimodal categorization. They specifically focused on stressful surroundings for visually impaired mobility, highlighting the versatility of these methods in many situations^91^. Burger and van den Heever made more advancements in improving EEG data by suggesting a technique to remove electrooculogram (EOG) artifacts, which is essential for guaranteeing the precision of emotional state assessments^92^. In addition, Akhand et al. (2023) proposed an updated method for recognizing emotions using EEG data, which improves the connection of information and provides breakthroughs in identifying and interpreting emotional states^93^.

Within the field of interior architecture education, there is a growing focus on studying the impact of emotional states on design processes. This involves using both traditional and digital methods to explore this area of research. The combination of brain-computer interfaces and emotion identification algorithms has facilitated the monitoring of students' emotional experiences during the design phase. It also identifies variations in emotional states based on the use of design tools and teaching methods. Moreover, the study recognizes the need to deliver research findings in a way that is simply understandable, highlighting the significance of conveying intricate emotional data collected from EEG in accessible words to enhance overall understanding.

The findings have wide-ranging ramifications beyond academics, providing practical insights into customizing teaching techniques in interior architecture according to emotional cues and tool choices. By combining the findings from the studies mentioned earlier^90–93^ with the current research, we can gain a thorough understanding of how EEG-based emotion recognition improves educational practices and highlights the possibility of using brain-computer interfaces in educational settings to enhance learning experiences based on emotional states.

We believe that with the assistance of brain-computer interfaces, such as the EEG devices used in this research, we will be able to collect more data, and with the assistance of data clustering, we will be able to come to different conclusions about the emotion recognition of the students and other groups, which will allow us to improve the quality of both education and life.

## Data Availability

The datasets generated and/or analyzed during the current study are available in the Github repository. https://github.com/sevgisengulayan/Neurocognitive-Responses-to-Spatial-Design-Behaviors.
